# Effects of Structural and Microstructural Features on the Total Scattering Pattern of Nanocrystalline Materials

**DOI:** 10.3390/nano12081252

**Published:** 2022-04-07

**Authors:** Nicola Dengo, Norberto Masciocchi, Antonio Cervellino, Antonietta Guagliardi, Federica Bertolotti

**Affiliations:** 1 Dipartimento di Scienza e Alta Tecnologia & To.Sca.Lab, Università dell’Insubria, via Valleggio 11, 22100 Como, Italy; nicola.dengo@uninsubria.it (N.D.); norberto.masciocchi@uninsubria.it (N.M.); 2 Swiss Light Source, Paul Scherrer Institut, 5232 Villigen, Switzerland; antonio.cervellino@psi.ch; 3 Istituto di Cristallografia & To.Sca.Lab, Consiglio Nazionale delle Ricerche, via Valleggio 11, 22100 Como, Italy; antonella.guagliardi@ic.cnr.it

**Keywords:** Debye scattering equation, atomic pair distribution function, quantum dots

## Abstract

Atomic- and nanometer-scale features of nanomaterials have a strong influence on their chemical and physical properties and a detailed description of these elements is a crucial step in their characterization. Total scattering methods, in real and reciprocal spaces, have been established as fundamental techniques to retrieve this information. Although the impact of microstructural features, such as defectiveness of different kinds, has been extensively studied in reciprocal space, disentangling these effects from size- and morphology-induced properties, upon downsizing, is not a trivial task. Additionally, once the experimental pattern is Fourier transformed to calculate the pair distribution function, the direct fingerprint of structural and microstructural features is severely lost and no modification of the histogram of interatomic distances derived therefrom is clearly discussed nor considered in the currently available protocols. Hereby, starting from atomistic models of a prototypical system (cadmium selenide), we simulate multiple effects on the atomic pair distribution function, obtained from reciprocal space patterns computed through the Debye scattering equation. Size and size dispersion effects, as well as different structures, morphologies, and their interplay with several kinds of planar defects, are explored, aiming at identifying the main (measurable and informative) fingerprints of these features on the total scattering pattern in real and reciprocal spaces, highlighting how, and how much, they become evident when comparing different cases. The results shown herein have general validity and, as such, can be further extended to other classes of nanomaterials.

## 1. Introduction

Colloidal semiconductor nanocrystals, also known as quantum dots (QDs), show excellent size- and shape-tunable optical and electronic properties due to a strong quantum confinement effect, as well as low-cost solution processability and surface functionalization [1].

Among colloidal QDs, binary III-V and II-VI semiconductors are most promising in terms of applications in displays [2,3] and solar cells with relatively high certified power conversion efficiencies [4,5]. Thanks to the seminal work of Murray, Norris, and Bawendi [6] and the following developments in the late 1990s and early 2000s, great progress has been made in the synthesis of colloidal QDs with unprecedented monodispersity, thus ensuring very narrow and tunable emission spectra [7].

Most III-V and II-VI semiconductors can crystallize in two polymorphs, as zincblende (ZB) and wurtzite (WZ) type-structures, where the cubic or hexagonal packing of layers is characterized by the same tetrahedral units MX_4_ (where M = metal of the XII/XIII groups and X = pnictide/chalcogenide) in the staggered (ZB) or eclipsed (WZ) configurations. This small difference between the two structures makes the occurrence of planar defects, in particular, of basal-plane stacking faults (SFs), a highly probable event, the density of which mainly depends on the ionicity of the M-X chemical bond [8].

The most-studied among luminescent binary semiconductors is cadmium selenide (CdSe), which is indeed characterized by very low SFs energy at the bulk scale (14 ± 5 mJ m^−2^) [9], leading to relatively high SFs densities.

At the nanoscale, where the formation of SFs can be strongly influenced by surface energy contributions [10,11], the possibility of ZB and WZ packings coexisting in the same nanocrystal makes the phase purity control a difficult task [12]. Our recent work on ZB CdSe QDs and its quantitative picture of planar defects’ dependence on nanocrystal size, based on a sophisticated atomistic model, is further confirmation of such complexity [13].

Accordingly, considering the huge impact of structural defects on the electronic properties of QDs [14,15,16,17,18], as well as their size and morphology playing a pivotal role in driving quantum confinement effects [19,20,21,22,23], developing experimental and computational methods able to retrieve structural and microstructural information on semiconductors at the nanoscale is of utmost importance. Transmission electron microscopy (TEM) is by far the leading technique in this field, being limited, however, in the statistical point of view and able to provide 2D information only, on selected particles, which might not be representative of the entire ensemble [24]. Imaging techniques often fail in precisely determining QDs morphology, due to resolution limits and the presence of capping ligands at the nanocrystals’ surface, generating blurry particle contours [25]. Additionally, precise structural information cannot be derived with these methods. 

The common modeling technique based on the Rietveld method (which is based on the original Braggs’ approach to intensity calculation from the atomic content of a unit cell) implies that nanoparticles are described simply as if they were just a small portion of a much larger massive material. To deal with NCs scattering traces, analytical profile functions are typically used in convolutory mode to model the size and shape of the nanocrystalline domains and the diffuse scattering arising from nanosize, shape, and/or defects is completely disregarded (i.e., hidden by fitting the continuous “background” trace through phenomenological polynomial functions) [26].

In this context, in the last decade, new approaches have emerged as superior methods for the structural analysis of nanoparticles: the wide angle X-ray total scattering (WAXTS) techniques [27]. Total scattering means that all (structure-relevant) scattering from the sample is collected and properly modeled, including both Bragg peaks, originating from nanocrystals’ atomic periodicity, and diffuse components, arising from size- and defects-induced effects [28]. These methods operate both in reciprocal (the so-called Debye-scattering-equation-based methods, DSE) and direct (pair distribution function analysis, PDF or *G*(*r*) function) spaces. Within the DSE-based approach, we have developed a thorough methodology that, starting from atomistic models of nanocrystals (thus including size, shape information, and many possible sources of structural defects), allows the computation of the total scattered intensity from a randomly oriented ensemble (both powders or colloidal suspensions) [29]. This computationally heavy approach is made feasible thanks to the exploitation of symmetry elements within the structure of nanocrystals, thus reducing the number of unique distances to be computed, and a fast implementation of the DSE, based on equispaced, sampled, interatomic distances (instead of the true ones), encoded in suitable databases [30]. As demonstrated by us [13,31,32], this approach can be easily extended to the simultaneous modeling of the small-angle (SAXS) and wide-angle (WAXS) regions of the total scattering pattern, thus allowing the characterization of structure, morphology, and defectiveness of nanocrystals within a unified modeling approach. 

Due to the powerful possibility of modeling both Bragg and diffuse scattering starting from atomistic models, the DSE-based approach has indeed been widely used in the literature to characterize size-, shape-, and defects-induced effects on the X-ray scattering pattern of nanocrystals, highlighting, for example, the possibility of deriving specific features arising from anisotropic particles morphology [24,33] and planar defects, especially dealing with metallic clusters [26,34].

On the other hand, PDF is a real-space total scattering method, requiring a maximized *Q* experimental value (where *Q* = 4*π* sin*θ*/*λ* is the magnitude of the scattering vector) to reduce truncation errors upon *sine*-Fourier transforming the total scattering profile [35]. One of the most popular approaches to PDF relies on a “real-space Rietveld-based method” (also called small-box modeling) in which the fitting of the experimental *G*(*r*) function is achieved upon varying atomic parameters within the unit cell (or a chosen supercell), eventually convoluted to a dampening function to account for the discrete size of the particles (form factor) and/or instrumental resolution (usually treated as a pure Gaussian contribution) [36]. For these reasons, the main fingerprints of structural and microstructural features of atomistic models of nanocrystals on the PDF are not clearly derived in the current literature. Nevertheless, some recent attempts to retrieve interlaced structural and morphological features from PDF data of nanocrystals (mainly metals and oxides) have been conducted using a numerical approximation of the nanoparticles form factor [37].

In this article, we demonstrate that total scattering methods can be used as powerful tools to obtain accurate information on particles from the atomic-to-nanometer length scales. In performing this exploration we have chosen, as benchmark nanocrystals, CdSe QDs, one of the most-studied model systems for investigating a wide range of optoelectronic and chemical processes [38]. 

Considering size and shape polydispersity, as well as different kinds of structural defects within a unified atomistic modeling approach (according to the DSE-based methodology), we explore their main fingerprints on the total scattering pattern of CdSe QDs in reciprocal space. Furthermore, we analyze the same features after sine-Fourier transforming of the DSE patterns, to compare the information retrievable in the two (real and reciprocal) spaces, also considering the different experimental conditions required by the two techniques. The model parameters are chosen to be realistic for CdSe QDs showing a highly predominant ZB cubic structure, as derived by a previous in-depth DSE-based study [13]. 

## 2. Methods

### 2.1. The Debye Scattering Equation (DSE) Approach

The Debye scattering equation (DSE) [39] provides the total scattered intensity (i.e., the average differential elastic cross-section) of a randomly oriented object from the distribution of interatomic distances between atomic pairs, within the sample:

(1) $$I\left(Q\right)=\overset{N}{\underset{j=1}{\sum }}f_{j}{\left(Q\right)}^{2}o_{j}{}^{2}+2\overset{N}{\underset{j>i=1}{\sum }}f_{j}\left(Q\right)f_{i}\left(Q\right)T_{j}\left(Q\right)T_{i}\left(Q\right)o_{j}o_{i}\frac{sin\;\left(Qd_{ij}\right)}{\left(Qd_{ij}\right)}$$

where Q=4πsinθ/λ is the magnitude of the scattering vector, *λ* is the radiation wavelength, *f_i_* is the X-ray atomic form factor of an element *i*, *d_ij_* is the interatomic distance between atoms *i* and *j*, *N* is the total number of atoms, and *T* and *o* are the thermal atomic displacement parameter and the site occupancy factor associated with each atomic species, respectively. The first summation accounts for the contribution of the zero distances of each atom from itself, whereas the second one includes the non-zero distances between pairs of distinct atoms. 

The DSE simulations of CdSe ZB QDs are carried out using the Debussy suite of programs [29], following a bottom-up approach. The CdSe ZB unit cell (*a* = 6.0400 Å) [13] is used as a building block to generate populations of atomistic models of QDs of increasing sizes. Bivariate (square *D_4h_* prisms, with two independent growth directions, *L_a_* = *L_b_* and *L_c_*) populations of nanocrystals are mainly used for the DSE simulations, along with monovariate (spherical shape, with progressively growing diameter, *D*) populations for a few selected cases. According to the Debussy strategy, for each population, sampled interatomic distances [30] of all pertinent clusters are encoded in databases and used to compute the DSE. Each cluster of the population is weighted by the appropriate fraction, according to a log-normal distribution of sizes, whose mean and standard deviation values are given, for all the models, in Table S2.

#### 2.1.1. The Instrumental Profile

Different experimental setups affect both the angular resolution and the peak shapes of the diffraction profile [40,41]. The characteristic shape of the observable peaks, in principle, can be computed by considering the geometry of the measurement, the detector used, the optics of the instrument, the sample holder, etc., according to the so-called fundamental parameters approach [42]. Alternatively, it is possible to derive the instrumental profile by adopting a convolutional approach with a phenomenological (analytical) function, describing the instrumental contribution to peak shape and width and its angular dependence as derived from a reference measurement. 

Within this approach, the instrumental resolution function (IRF) used here (and convoluted with the model DSE) is a pseudo-Voigt function (i.e., a linear combination of two—one Gaussian, G(θ), and one Lorentzian, L(θ)—functions):

(2) PV(θ)=ηL(θ)+(1−η)G(θ)

characterized by the following angular dependence of the full width at half maximum (*FWHM*):

(3) FWHM(θ)=ha+hbtanθ+hc/cosθ

where *ha*, *hb*, and *hc* are real numbers, refined using TOPAS v3.0 [43] against reference materials collected in the experimental conditions of interest, and are reported in Table S1. For the sake of simplicity, the mixing parameter, *η* (0≤η≤1), is kept constant in the whole angular range (Table S1). 

To account for realistic experimental conditions for each technique (as further detailed in Section 3.1), an appropriate level of noise, distributed according to a Poisson statistic [44], is added to the DSE simulations, as detailed in the Supporting Information.

#### 2.1.2. Stacking Disorder in CdSe ZB QDs

Atomistic models of prismatic CdSe nanocrystals are built by stacking atomic monolayers along the [111] direction of the cubic unit cell (in the trigonal setting: $a_{trig}=-\frac{1}{2}a_{cub}+\frac{1}{2}b_{cub}$, $b_{trig}=-\frac{1}{2}b_{cub}+\frac{1}{2}c_{cub}\;$and $c_{trig}=a_{cub}+b_{cub}+c_{cub}$). 

Stacking disorder along [111] is introduced by randomly assembling monolayers of Cd-Se dumbbells (aligned with [111], with a fixed bond distance of 2.63 Å), following the formalism proposed by Kakinoki [45]. 

**Figure 1:**
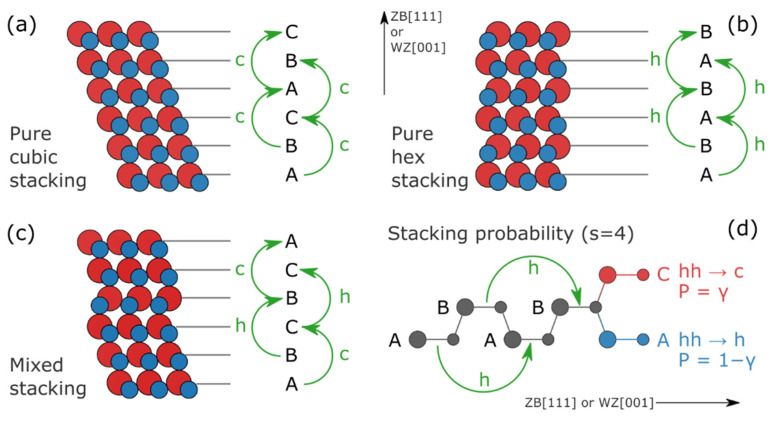
Sketches of ZB (**a**), WZ (**b**), and mixed stacking (**c**) structures, highlighting the different {ABC}_n_ stacking sequences along [111] (cubic, *c*) and {AB}_n_ stacking sequences along [001] (hexagonal, *h*). (**d**) An example of a stacking sequence associated with an SF probability parameter (P = γ and the related P = 1 − γ) within the *s* = 4 formalism.

In a close-packed faulted structure, three types of atomic layers can be stacked along the main growth direction ([111] in a ZB-type structure and [001] in a WZ-type), named *A*, *B*, and *C*, according to the different atomic coordinates in the plane perpendicular to [111] (ZB) or [001] (WZ), as shown in Figure 1a–c. The final arrangement of layers (along [111] in this work) is determined by a Markov chain sequence: in the simplest example, each layer influences the occurrence of the next one only (no “memory effect”, *Reichweite* parameter, *s* = 1) [46], considering also that a sequence of equal layers (e.g., *AA*) is forbidden.

As described in ref. [13], for CdSe QDs a longer correlation length is implemented by using *s* = 4, meaning that the occurrence of an atomic layer depends, as a stochastic event, on the preceding 4 layers (Figure 1d). According to this formalism, for close-packed structures (for which the *AA*/*BB*/*CC* sequences are forbidden), the number of different configurations allowed for an *s*-layers sequence is *R* = *6s−3*.

Therefore, after redefining an *ABC* sequence (or any equivalent one in which the third layer differs from the first one) as *c* (i.e., cubic), and an *ABA* concatenation (having the third layer equal to the first one) as *h* (i.e., hexagonal), four independent SFs probability parameters are considered:

*α* = P(X_n+1_ = c | X_n−1_ = c, X_n_ = c), e.g., *ABCA*→*B*

*β* = P(X_n+1_ = *c* | X_n−1_ = *h*, X_n_ = *c*), e.g., *ABAC*→*B*

*γ* = P(X_n+1_ = *c* | X_n−1_ = *h*, X_n_ = *h*), e.g., *ABAB*→*C*

*δ* = P(X_n+1_ = *c* | X_n−1_ = *c*, X_n_ = *h*), e.g., *ABCB*→*A*

where *α, β, γ, δ* span from 0 to 1 and are given as input parameters in the assembly of the sequences of layers. 

A matrix *N_lay_* ✕ *N_seq_* is therefore generated, with *N_lay_* being the number of CdSe layers along [111] and *N_seq_* the number of sequences for each input parameter, representing the different possible configurations. *N_lay_* depends on the size of the cluster considered, while *N_seq_* is chosen to be equal to 50 for all databases (based on previous tests, details can be found in ref. [13]).

Multiple databases of sampled interatomic distances are obtained in this way, according to bivariate populations of CdSe QDs (two growth directions, *L_a_* = *L_b_* and *L_c_*), following the Debussy strategy. 

The final databases for each cluster of the population characterized by the same *α*, *β*, *γ*, *δ* are then computed as linear combinations of the equispaced pseudomultiplicities (associated with the sampled interatomic distances) of the *N_seq_* generated, from which the DSE is computed, as detailed in Section 2.1. 

The stacking faults input parameters used in this work are given in Table S3 for all the models. 

In order to provide SFs average quantification, independent from the model used, an SF% is computed as: $SF\%=\left[\overset{N}{\underset{i=1}{\sum }}{\left(\frac{A_{h}}{A_{h}+A_{c}}\right)}_{i}\frac{V_{i}\xi_{i}}{\sum_{j=1}^{N}V_{j}}\right]\times 100$, where *N* is the total number of clusters in the population, *A_h/c_* is the total area of the *h*/c layers of the *i*-th cluster, *V_i_* its volume, and ξ its number fraction, according to the bivariate log-normal distribution function implemented for the DSE simulation. Therefore, the SF% is here considered as the prevalence of (wrong) hexagonal stackings within the cubic reference structure.

### 2.2. The Atomic Pair Distribution Function (PDF) Method

As detailed in Section 2.1, the coherent scattered intensity from a randomly oriented object can be computed by using the DSE (Equation (1)). 

The self-scattering term of the DSE, containing only *i* = *j* elements, can be redefined as the average of the squared scattering factors:

(4) $$\frac{1}{N}\overset{N}{\underset{j=1}{\sum }}f_{j}^{2}\left(Q\right)=\langle f^{2}\rangle \left(Q\right)$$

where *N* is the total number of atoms in the system and Q=4πsinθ/λ. 

By dividing total scattered intensity computed through the DSE by the self-scattering term, it is possible to calculate the so-called total scattering structure function [47]:

(5) $$S\left(Q\right)=\frac{I\left(Q\right)}{\langle f^{2}\rangle \left(Q\right)}$$

Once the S(Q) is obtained, the atomic PDF, G(r), can be computed, as sine-Fourier transform of F(Q)=Q[S(Q)−1] (reduced total scattering structure function):

(6) $$G\left(r\right)=\frac{2}{\pi }\int_{Q_{min}}^{Q_{max}}Q\left[S\left(Q_{i}\right)-1\right]\sin QrdQ$$

According to this workflow, implemented in the Debussy suite of programs [29], the S(Q) is computed from each DSE pattern (with the proper *IRF* and signal-to-noise ratio) and properly rescaled in order to obtain $\underset{Q\to \infty }{\lim}F\left(Q\right)=0$ (Figure S1). 

The values of *Q_min_* and *Q_max_* for each computation are reported in Table S1 and they are chosen to avoid oscillations coming from the small-angle scattering signal (*Q_min_*) and to recover the *Q*-space resolution of the experimental data (*Q_max_*) [48], depending on the selected instrumental setup (as detailed in Section 3.1). 

On the other hand, the PDF is sampled according to a grid of mesh *dr* = 0.01 Å, as is common practice to generate smooth G(r) signals; the comparison between a PDF obtained using *dr* = 0.01 Å and the optimal sampling step according to the Nyquist–Shannon theorem ($dr=\pi /Q_{max}$) [49] is reported in Figure S2, showing that the oversampling does not generate any artifact in the computed G(r).

## 3. Results and Discussion

### 3.1. On the Instrumental Resolution Function

To compare different modeling aspects in real and reciprocal spaces by generating ad hoc synthetic datasets, the pertinent experimental setup must be considered. 

**Figure 2:**
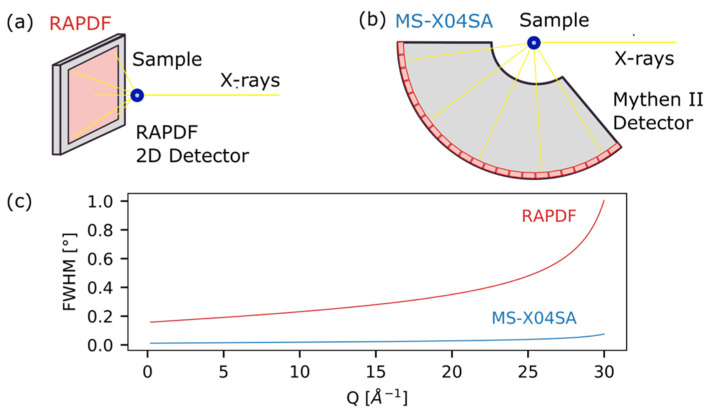
Schematics of the experimental setups employed for (**a**) RAPDF acquisition mode for high-*Q* X-ray total scattering data (PDF analysis) and (**b**) high angular resolution datasets, ensured by the implementation of a position-sensitive 1D array detector, employed for reciprocal space data analysis. (**c**) Full width at half maximum (FWHM) vs. *Q* for the two experimental setups reported in (**a**) (IRF: *ha* = 0.07807, *hb* = 0.20620, and *hc* = 0.07882; *η* = 0.15729) and (**b**) (IRF: *ha* = 0.01130, *hb* = 0.01990, and *hc* = 0.00010; *η* = 0.35903), showing the poorer angular resolution for the RAPDF acquisition mode and the angle-dependence of both instrumental resolution functions.

In this view, for the simulations reported in the next sections of the manuscript (Section 3.3 and Section 3.4), we consider two of the most common setups implemented for PDF and DSE data collections: the so-called “rapid-PDF” (RAPDF) acquisition mode (Figure 2a) [50], and a high angular resolution setup combined with a high signal-to-noise ratio (SNR), ensured by employing a position-sensitive 1D detector (Figure 2b) [27,51].

One of the main issues related to the sine-Fourier transform of total scattered intensity is the occurrence of truncation errors due to the inevitable *Q_max_* experimental limit, resulting in unphysical, high-frequency oscillations in the *G*(*r*) [28,52].

Therefore, to maximize *Q_max_*, as well as SNR at high *Q*, standard PDF data collections usually employ an RAPDF setup, combined with high operating energies (>50 keV). This experimental condition, achieved with a large area detector placed very close to the sample (150–300 mm), allows a large *Q*-range to be collected (in Debye Scherrer geometry) in a single shot (*Q_max_* typically > 20 Å^−1^) [53]. 

However, due to the very short sample-to-detector distance and to the characteristics of area detectors, this experimental setup is associated with poor angular resolution. In Figure 2c, the full width at half maximum (FWHM) of an instrumental resolution function (IRF) computed from a reference material usually employed for PDF experiments (polycrystalline nickel) is compared with a line position and shape standard (Silicon NIST 640c) from a high-resolution setup. The nickel powder dataset was collected at the 28-ID-1 PDF beamline at NSLS-II (Brookhaven National Laboratory, Upton, NY) according to an RAPDF acquisition mode (69 keV, *Q_max_* = 23Å−1). The silicon NIST 640c standard experimental data were collected at the MS- X04SA beamline of the Swiss Light Source (SLS, Paul Scherrer Institut, Villigen, CH), implementing a 1D silicon microstrip array detector (MYTHEN-II) [54], covering simultaneously up to 155° of *2θ*, with a sample-to-detector distance of 760 mm (17 keV, *Q_max_* = 19Å−1). 

Details on the IRF parameters, refined against experimental data, are reported in Figure 2 and Table S1.

It is worth noticing that the FWHM of the IRF of the RAPDF@NSLS-II measurements has not only a larger constant term with respect to the SLS one, but also a larger angular dependence, while it has a slightly smaller mixing parameter, *η*. Its small value (0.16) indeed indicates that the instrumental profile of the RAPDF@NSLS-II setup is dominated by the Gaussian contribution, which makes the pure Gaussian approximation, implemented in popular PDF software such as PDFgui [36], quite reliable. 

As illustrated in the next section, the poor angular resolution characterizing RAPDF@NSLS-II experiments hampers reliable nanocrystals size estimation when samples’ coherent domains exceed ∼7 nm, as a consequence of the intrinsic *G*(*r*) dampening at large *r*-values originating from finite (and large) peak widths in Q space (mimicking size-induced broadening).

All the simulations reported in the next sessions (3.2–3.4) are performed considering the experimental conditions (in terms of *Q_max_*, IRF, and SNR) associated with the two setups: RAPDF for PDF simulations and the MS-X04SA conditions for the DSE ones, detailed in Table S1. 

### 3.2. Influence of Nanocrystals Size and Size Distribution on Total Scattering Patterns

Beyond structural features, X-ray diffraction patterns also contain valuable microstructural information. According to conventional Rietveld-like methods of analysis and theoretical analyses, the sample diffraction profile is conceived as a convolution of a Gaussian and a Lorentzian function, where the first component is largely due to microstrain, while the latter is normally taken as a consequence of the finite size of crystalline domains [55]. If size-monodisperse samples are considered, this method works properly when also dealing with very small QDs, as demonstrated elsewhere [27]. Nevertheless, this traditional approach, extracting size information from Bragg peaks only, completely neglects the diffuse scattering between and below them, also arising from the finite size of (nano)crystalline domains. Moreover, a plethora of other common features in very small QDs, such as size dispersion and morphological effects, structural defectiveness, and other kinds of static disorder, are normally, though not always, disregarded [27]. 

**Figure 3:**
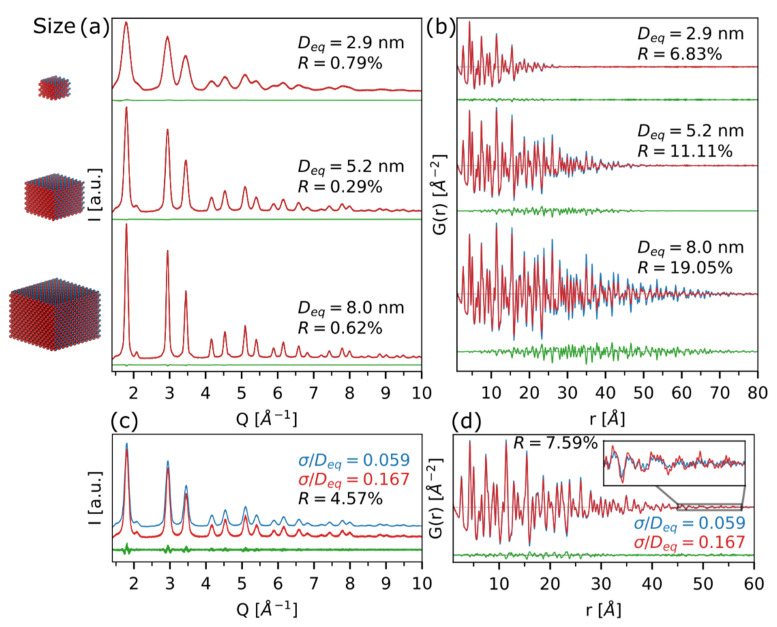
Simulations in reciprocal (DSE, first column) and real (PDF, second column) spaces for CdSe atomistic models, with different sizes (**a**,**b**) and size dispersions at a constant nanocrystal size (5.2 nm) (**c**,**d**). In (**a**,**b**), the difference trace (green line) is computed against the ideal simulation (without IRF convolution), while in (**c**,**d**), it is the difference between the monodisperse population (blue line) and the polydisperse one (red trace), both with the proper IRF convolution. An agreement factor, computed as $R=\left(\sqrt{\frac{\sum_{i=1}^{N}{\left(I_{r}-I_{w}\right)}^{2}}{\sum_{i=1}^{N}I_{r}^{2}}}\right)\times 100\;$ (where *I_r_* is the reference simulation, and *I_w_* the work model) is also reported. Further details on the size and shape of CdSe atomistic models are given in Table S2.

In Figure 3a,c, the role of size and size distribution on the WAXTS DSE pattern of CdSe QDs (ZB structure) is highlighted: size-induced peak broadening and increasing of diffuse scattering components upon downsizing are clearly visible in Figure 3a, while the size dispersion modification (from a strict monodisperse up to a slightly polydisperse population of nanocrystals) is mainly influencing Bragg peaks’ tails (Figure 3c). Therefore, from the reciprocal space patterns, it is possible to disentangle the effects coming from (nano)crystalline domains lowering/enlargement and nanoparticles size distribution modifications.

Moving to real-space simulations, size and size dispersion effects are shown in Figure 3b,d, respectively. According to literature reports, the average coherence length of (nano)crystalline domains can be estimated in the direct space by the dampening properties of the PDF signal [53]. 

The simplest method to perform this calculation is measuring the *r*-value at which the *G*(*r*) signal is fading away, since no interatomic distances falling at *r* > *D* are possible. Within the so-called small-box modeling approach, implemented in some popular software, such as PDFgui [36], the envelope function, dampening the *G*(*r*) at high *r*, can be multiplied to the reference *G_bulk_*(*r*), giving the final attenuated PDF. The envelope function in the most standard case is a spherical form factor but can be in principle replaced with other analytical expressions [56] (or even numerical approximations) [37] accounting for different morphologies. In Figure 3b, the vanishing of the *G*(*r*) is quite consistent with the CdSe QDs nominal sizes (*D_eq_*, diameter of the spherical cluster of equivalent volume) used for the simulations of 2.9 nm and 5.2 nm clusters, while for the largest system it occurs at *r* ∼ 6.5 nm, despite the 8.0 nm size of the original QD. In real materials, this apparent lower size extracted from the *G*(*r*) could be a consequence of many sample-related effects, such as surface amorphization or occurrence of structural defects, lowering the “size” of the coherent domains (as detailed in Section 3.4); this could therefore be easily misinterpreted. In the example shown in Figure 3b, the 8.0 nm QDs signal is dampened because of the poor *Q*-resolution of the RAPDF experiment, making unreliable any size estimation above the resolution limit. This value can be roughly estimated, e.g., by looking at the *G*(*r*) of the highly crystalline material used as reference (in this example, polycrystalline Ni, shown in Figure S3). 

Considering QDs size dispersion effects (Figure 3d), the strict monodispersed condition (5.8%), applied to the selected 5.2 nm CdSe cluster, quite usual for III-V and II-VI semiconductors [13], does not significantly modify the PDF trace; on the other hand, a slightly larger size dispersion (23.4%), not so uncommon for many other semiconductor nanocrystals (such as lead halide perovskites) [57], might determine a sample size overestimation, up to ∼5.8 nm, if not properly accounted for in the model description. Therefore, for an accurate size determination, polydispersity should also be considered in building models for real-space structural analysis. 

### 3.3. Extracting Morphological Features from Wide-Angle X-ray Total Scattering

**Figure 4:**
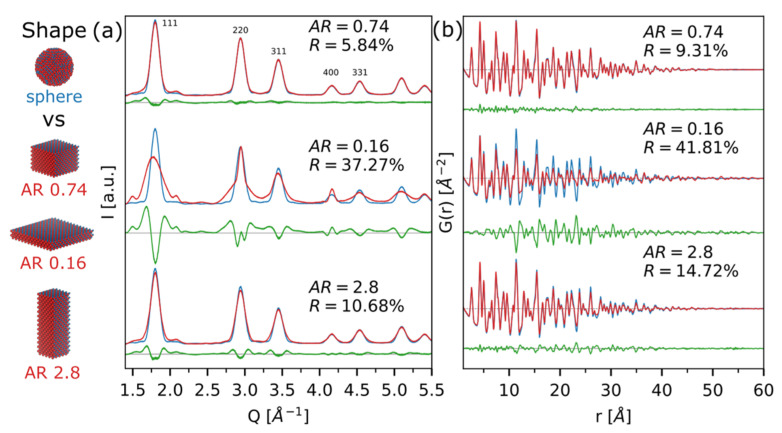
Comparison between simulations of spherical CdSe QDs (*<D>* = 5.2 nm; σ/*<D>* = 7%, blue line) and different square prism morphologies with aspect ratios (*AR* = *L_c_*/*L_a_*) 0.7, 0.2, and 2.8 (red lines), both as DSE (**a**) and PDF (**b**). Differences against the reference spherical model are shown as green traces at the bottom of each plot, together with the corresponding agreement factors, *R*. Further details on the size and shape of CdSe atomistic models are given in Table S2.

Colloidal QDs such as CdSe are ideal model systems for nanoscience, due to the extraordinary control over their size and shape that has been developed through the years [7]. However, beyond highly controlled synthetic procedures allowing precise tuning of particles’ sizes and shapes, it is equally important to develop ensemble characterization methods able to properly quantify these features and to relate them with QDs properties. Despite being the leading technique in the field, TEM size and shape determination of very small nanocrystals, such as QDs, is generally hampered by difficulties in particle/background evaluation and other contrast-related issues [25]. Therefore, scattering methods represent a valid alternative due to the intrinsic microstructural information stored in both small- and wide-angle regions of the total scattering patterns of nanocrystals. In Figure 4, we explore this possibility considering the wide-angle region only, this being also the carrier of structural information. 

Figure 4 showcases the comparison between a reference spherical morphology (*<D>* = 5.2 nm; σ/*<D>* = 7.0%) and alternative square prisms (*L_a_ = L_b_ ≠ L_c_*) with aspect ratios (*AR* = *L_c_*/*L_a_*) 0.7 (as detected in CdSe ZB experimental data) [13], 0.2 (plate-like), and 2.8 (rod-like), in reciprocal (Figure 4a) and real (Figure 4b) spaces. 

To focus on the shape-induced differences in the wide-angle total scattering region, all the populations of nanocrystals used for performing the simulations have the same average volume (78 nm^3^) and relative size dispersions along the two growth directions (*σ/L_a_* = 6.9%; *σ/L_c_* = 10.7%), comparable to the ones detected on real CdSe samples [13]. The effect of slightly modifying the morphology (from a spherical to a square prim with *AR* = 0.7) is very tiny both in real and reciprocal spaces, hidden by size-dispersion-induced effects on peak tails in the DSE simulation and is perhaps undetectable unless complementary SAXS techniques are considered [13]. On the other hand, more anisotropic morphologies are clearly modifying the main features of the DSE simulations (Figure 4a), especially when a very short axis is included in the model (*L_a_* = *L_b_* = 7.7 nm; *L_c_* = 1.3 nm, *AR* = 0.2), determining the occurrence of broader components together with sharper ones. Anisotropic morphologies in the *G*(*r*) (Figure 4b) are detectable as an increase of the maximum coherence length with respect to the reference spherical simulation (fading at 52 Å) and with a different slope of the *G*(*r*) dampening with *r*. 

**Figure 5:**
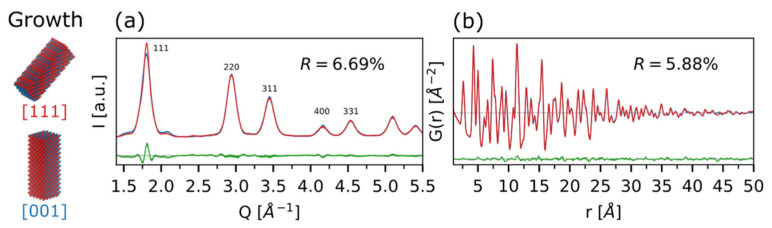
Influence of the growth directions ([100]: red line; [111]: blue trace) on the CdSe simulations in real (**a**) and reciprocal (**b**) spaces. Bragg peaks indices are reported for the DSE simulations in order to highlight the selectivity on their intensities/widths of the two models. Differences between the two growth directions ([111] and [100]) are shown as green traces at the bottom of each plot, together with the corresponding agreement factors, *R*.

Considering *AR* = 2.8 as a reference (*L_a_* = *L_b_* = 3.0 nm; *L_c_* = 8.4 nm), we also explore the influence on the WAXTS pattern of different growth directions of CdSe ZB QDs, these being [100] preferred for ZB structures, while [111] can be considered a plausible occurrence upon intergrowing with the WZ-like hexagonal polymorph, due to the mutual orientations of the two crystal structures (Figure 5). The two growth directions are immediately distinguishable in the DSE patterns (Figure 5a) due to the sharpening of 111 and of 400 peaks (200 being of very low intensity in CdSe), for the [111] and [100] growth directions, respectively. In real space (Figure 5b), the (relative) differences between the two morphologies are maximized in the high-*r* region (*r* > 30 Å), being however difficult to reconcile the tiny *G*(*r*) variations with any evident growth direction. Moreover, this spatial region is also highly influenced by other sample- or instrumental-related effects, making morphological features difficult to infer from PDF traces only; this is also the case for very small QDs, especially when an RAPDF data collection strategy is employed. 

### 3.4. Structural Defectiveness in Real and Reciprocal Spaces

Stacking disorder is reported in a large variety of materials, from layered compounds [58,59,60,61] to close-packed metals [34,62,63,64], alloys [65], and semiconductors [66,67,68,69,70,71]. Generally speaking, it arises because the different ways the layers stack on top of each other possess close (enough) energetics. In close-packed materials, it can be (qualitatively) described as a dense intergrowth of two polymorphs (f.c.c. and h.c.p.), generating an aperiodic stacking sequence along the growth direction ([111] in the cubic and [001] in the hexagonal phases); the reference modeling approach is the formalism proposed by Kakinoki [45], also defined by the *Reichweite s* = 4 [72], meaning that a single atomic layer influences the stacking arrangement up to the fourth neighbor (through four independent probability parameters α: *cc*→*c*; β: *hc*→*c*; γ: *hh*→*c*; δ: *ch*→*c*). 

The implementation of this formalism within the DSE-based modeling approach has recently allowed the quantification of CdSe QDs SFs density, and a detailed reconstruction of particles’ morphological details, within a unified atomistic model [13]. Previous attempts were made employing real-space modeling, which were able to successfully retrieve average size information and SFs probability within a simplified *s* = 1 approach (no “memory effect” considered) [38,73,74,75,76]. On the other hand, the possibility of disentangling size-induced effects from defect-induced effects in the PDF (a challenging aspect in *Q*-space, since both features influence peak width) by performing *r*-dependent structural refinements has been considered fundamental to retrieving SFs probability up to *s* = 4 more straightforwardly and to even reveal overparameterization effects (*s* = 3 “synthetic data” fitted using an *s* = 4 model) [64].

**Figure 6:**
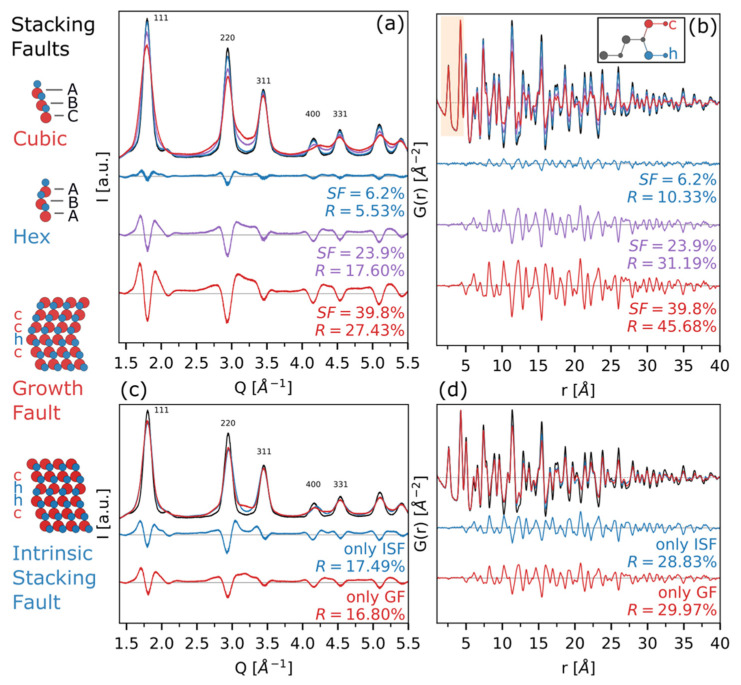
(**a**) DSE simulations of different SF% (6.2, 23.9, and 39.8) introduced in a CdSe population of prismatic nanocrystals with average sizes *L_a_* = *L_b_* = 4.7 nm (σ/*L_a_* = 6.9%) and *L_c_* = 3.5 nm (σ/*L_c_* = 10.7%). (**b**) Corresponding *G*(*r*), with the features in the low-*r* region assigned to interlayer interatomic distances (scheme in the inset), and highlighted with a shaded area. Effects on DSE peak width and position, due to growth (GF, α = 0.57, β = 1.00, γ = 1.00, δ = 1.00), and intrinsic SF (ISF, α = 0.85, β = 0.85, γ = 1.00, δ = 0.00) (**c**), and their corresponding sine-Fourier transform (**d**), at a constant SF% (∼23%). Difference traces and agreement factors against the unfaulted model are provided for all simulations. Further details on the SFs parameters used for these simulations are given in Table S3.

**Figure 7:**
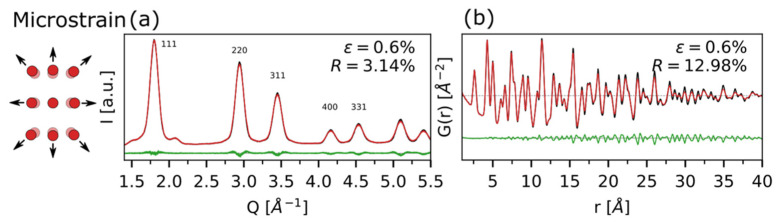
Isotropic microstrain effect, with *ε* = 0.6%, particularly effective on the peaks broadening in the high-*Q* region for the DSE simulation (**a**), and in the dampening of *G*(*r*) with *r* (**b**). The unstrained simulations (black lines) and the corresponding *R*-values are reported for comparison.

Considering the importance of obtaining a detailed structural and microstructural description (coupled with a thorough morphological characterization, already tackled in the previous paragraphs) of (nano)materials, we performed DSE/PDF simulations with different amounts and types of stacking disorder (Figure 6) and microstrain (Figure 7), the latter likely associated with non-uniform modulation of interplanar distances in the presence of SFs. Details on the models, implemented within the DSE-based approach and based on the *s* = 4 formalism, are found in the Method Section 2.1.2 and ref. [13].

Figure 6a,b show the DSE and PDF simulations, respectively, at increasing SF% on square prisms CdSe QDs of average sizes *L_a_* = *L_b_* = 4.7 nm (σ/*L_a_* = 6.9%) and *L_c_* = 3.5 nm (σ/*L_c_* = 10.7%). The increase of SFs percentage, from 0% up to 39.8%, clearly broadens Bragg peaks in the DSE simulations, in favor of a growing amount of diffuse scattering between and below them. At the highest SFs value (39.8%), features typical of the WZ phase appear at *Q*∼3 å^−1^ and in the higher *Q* region (*Q* > 4.4 å^−1^, Figure 6a). These peculiar features are complemented in *r*-space by the rise of new peaks corresponding to interatomic distances of the hexagonal polymorph in the range 10 å < *r* < 27 å, being the lower *r* region (*r* < 6 å) dominated by the intralayer features, and thus independent from the stacking sequence (Figure 6b). 

To verify the contribution of the so-called “memory effects”, in Figure 6c,d we worked at constant SFs percentage (∼23%) and we modified accordingly the four probability parameters (given in Figure 6 and Table S3) to generate faulted sequences of *…cchcc…* (growth fault only) and *…cchhcc…*(intrinsic SFs only), to be compared with the ideal ZB structure. The two types of SFs in percentage below 10% are well-known to induce Bragg peaks broadening only (growth fault, GF) or Bragg peaks broadening and shifts, depending on the reflection indices (intrinsic SF, ISF), and thus are distinguishable in reciprocal space [34]. 

When the number of planar defects increases up to the values shown in Figure 6c,d, new peaks appear in the *Q*-space of GF simulations only (clearly visible at *Q* = 3.25 å^−1^) due to the incipient development of a new periodicity, resembling one of the 6H_1_ polytypes, characterized by 1/3 of hexagonal components within the cubic structure as a periodic …*cch*… sequence (Figure 6c and Figure S4) [77]. Considering the sine-Fourier transform of the DSE, shown in Figure 6d, a clear fingerprint of the two types of defects is detectable: the major deviations from the unfaulted structure are detectable in the 10 å < *r* < 20 å for the GF-only simulation, while at higher *r*-values (*r* > 20 å), this structure recovers a higher degree of order (due to the incipient long-range ordering) with respect to the ISF model *G*(*r*). 

Therefore, since the SFs percentage is equal in the two cases, the “memory effect” of the structure (up to *s* = 4) is clearly preserved also in real space and (likely) the four SFs parameters are retrievable by using an appropriate modeling approach. 

In our recent work on CdSe QDs, beyond stacking faults’ defectiveness, an additional source of peak broadening in reciprocal space was found, following a *tan*(θ) dependence, and ascribed to modulation of interatomic distances along the stacking direction. The effect of a microstrain parameter ε = 0.6% (value optimized against experimental CdSe QDs data) [13] is shown in Figure 7a (*Q*-space) and Figure 7b (*r*-space). From DSE simulations (Figure 7a), the microstrain effect is clearly distinguishable from the SFs-induced one, causing peaks broadening only (without shifts), without any selectivity on reflection indices, in contrast to SFs that follow well-defined (i.e., vectorial) selection rules. A similar consideration can be conducted for the PDFs shown in Figure 7b, in which no additional peaks corresponding to the occurrence of new interatomic distances (belonging to WZ) appear once the microstrain is introduced in the model; nevertheless, a general coherence loss is retrieved as a consequence of the augmented peak broadening in the original DSE simulation.

## 4. Conclusions

To summarize, in this work we have shown how DSE can be used to simulate structural and microstructural features on very small QDs. By sine-Fourier transformation of the reciprocal space patterns, we have explored how size inhomogeneities, morphology, and structural defectiveness might also impress specific fingerprints on the corresponding PDFs, while the low angular resolution of the experimental setup, usually employed jointly for real-space data analysis, can be detrimental in properly identifying them. 

Among the most significant results derived, we quote: (i) the possibility of disentangling size and size dispersion in DSE patterns, as well as features coming from different growth directions in nanoclusters (coupled also with size dispersions effects); (ii) the concurrent sample- (size, size dispersion, and anisotropic morphologies) and instrumental-related effects on the PDF that might lead to misinterpretations of the results if not independently derived using other techniques; (iii) the persistence of “memory effects” related to stacking disorder, both in reciprocal and real spaces, thus potentially driving to a proper reconstruction of different stacking sequences in defective (nano)crystals (if an adequate model is employed); (iv) the feasibility of distinguishing between different kinds of structural defects in very small QDs, such as SFs and microstrain, both relying on the DSE and PDF methods of analysis. Moreover, their quantification is fully affordable for both methodologies (again under the assumption of a thoroughly implemented model) if an independent (ideally ensemble) technique is employed for size determination (such as SAXS) [13,78].

It is worth noting that the modeling approach usually employed for PDF analysis, based on the so-called “small-box” methodology, is clearly inadequate to describe all the structural and microstructural information reported in this work. 

A more thorough approach, such as the one based on atomistic models construction coupled to DSE computations, should be considered essential in this kind of study, which is indeed of utmost importance for a proper characterization of functional nanomaterials, such as CdSe QDs.

## Data Availability

The data presented in the manuscript are available upon reasonable request from the corresponding author.

## Supplementary Materials

The following supporting information can be downloaded at: https://www.mdpi.com/article/10.3390/nano12081252/s1, calculation of *G*(*r*) (Figures S1 and S2); limitations on average size determination using the RAPDF setup (Figure S3); effect of high SF% in the case of growth stacking faults (Figure S4); detailed information instrumental parameters (Table S1); size and size distribution (Table S2); stacking faults parameters (Table S3). References [13,50,54] are cited in the supplementary materials.

## Code Availability

The DebUsSy program suite is freely available at https://debyeusersystem.github.io.
