# Supplementary material for: Effects of Structural and Microstructural Features on the Total Scattering Pattern of Nanocrystalline Materials
Source: Nanomaterials (Basel). 2022 Apr 7;12(8):1252. doi: 10.3390/nano12081252 (PMC9030889; doi:10.3390/nano12081252)
Supplement: Supplementary file 1 [file nanomaterials-12-01252-s001.zip › nanomaterials-1654147-supplementary.pdf]

nanomaterials

MDPI

Supplementary Materials

Effects of Structural and Microstructural Features on the Total Scattering Pattern of Nanocrystalline Materials

Nicola Dengo [1], Norberto Masciocchi [1], Antonio Cervellino [2], Antonietta Guagliardi [3] and Federica Bertolotti [1,*]

[1] Dipartimento di Scienza e Alta Tecnologia & To.Sca.Lab, Università dell'Insubria, via Valleggio 11, 22100 Como, Italy; nicola.dengo@uninsubria.it (N.D.); norberto.masciocchi@uninsubria.it (N.M.)
[2] Swiss Light Source, Paul Scherrer Institut, 5232 Villigen, Switzerland; antonio.cervellino@psi.ch
[3] Istituto di Cristallografia & To.Sca.Lab, Consiglio Nazionale delle Ricerche, via Valleggio 11, 22100 Como, Italy; antonella.guagliardi@ic.cnr.it
* Correspondence: federica.bertolotti@uninsubria.it; Tel.: +390312386663

1. Instrumental Resolution Function and Signal to Noise Ratio

Two relevant instrumental setups were considered:

- Rapid-PDF (RAPDF). Based on data from the 28-ID-1 PDF beamline at NSLS-II (Brookhaven National Laboratory, Upton, NY).[1]
- High resolution setup, with 1D silicon microstrip array detector (MYTHEN-II). Based on data from the MS-X04SA beamline of the Swiss Light Source (SLS, Paul Scherrer Institut, Villigen, CH).[2]

The instrumental broadening was introduced in the model patterns in reciprocal space, by convoluting an Instrumental Resolution Function (IRF, i.e. a pseudo-Voigt), ad detailed in the main text.

The different Q ranges available by performing experiments with the two setups were also considered by choosing appropriate Q_{max} values, as reported in Table S1. For both setups, the same Q_{min} = 1.0964 Å^{-1} was chosen. To reproduce the different resolution capabilities, the total number of simulated data points was the same as the respective reference patterns.

Table S1. Parameters used to simulate different instrumental setups.

Instrumental setup	Q_{max} (Å^{-1})	Points	h_a	h_b	h_e	η	SNR
RAPDF	25	1481	0.07807	0.20620	0.07882	0.15729	100
MS-X04SA	19	33477	0.01130	0.01990	0.00010	0.35903	1400

Synthetic noise was added to the calculated scattering intensities (SNR = signal/noise), at a level that closely matches the typical noise observed in routine experiments performed at the reference beamline, as detailed in the following sections.

1.1. Determination of the Reference Signal-to-noise Levels

Reference noise levels were determined from diffraction patterns of CdSe suspensions in toluene measured at the two instrumental setups. The required data was available from previously performed experiments.

Due to the absence of a region on the diffraction pattern entirely free from sample signal, it was not possible to estimate an accurate noise level by simply calculating a single root mean square (RMS) value from a selected subrange of the pattern. This problem was solved by applying a high-pass filter to the experimental data $\boldsymbol{I_{exp}}$, to retain only high-frequency components that are compatible with instrumental noise.

$$\boldsymbol{I_{hp}} = \boldsymbol{highpass}[\boldsymbol{I_{exp}}]$$

Other complications arise from the effects of the data reduction procedure and the background subtraction that were applied to obtain the final patterns. Thus, the standard deviation of the distribution determining the level of noise is expected to be different from the one of a Poisson distribution, where $\sigma_i = \sqrt{I_{exp,i}}$.

Thus, the signal-to-noise ratio was here estimated as a vector instead of a single value. A vector of root mean square values was calculated from the filtered experimental intensities using a moving-average approach.

$$RMS_i = \frac{1}{n}\sqrt{\sum_i^n \left(I_{hp,i} - \frac{1}{n}\sum_{j=n-i}^{n+i} I_{hp,i}\right)^2}$$

The value of n (typically from 50 to 200) indicates the size of a subrange centered at the point i for the calculation of the moving average. Points at the two ends of the patterns that are incompatible with the calculation of the moving average were excluded from the calculation. Thus, the ***RMS*** vector has n points less than the original pattern. The obtained ***RMS*** vector can be used as an estimation of the noise affecting each data point of the experimental intensities (with the exclusion of the n points for which the RMS calculation was not possible at the pattern high and low-Q ends).

From this, a vector of reference signal-to-noise ratios (***S/N***) can be approximated as follows.

$$S/N_{exp,i} = \frac{signal_i}{\sigma_i} = \frac{max[\boldsymbol{I_{exp}}] - min[\boldsymbol{I_{exp}}]}{RMS_i}$$

Where the signal is approximated by the difference between the maximum and minimum intensity values. The noise value is instead represented by the standard deviation of the underlying distribution, estimated by the RMS. The calculated $\boldsymbol{S/N_{exp}}$ vector was used a reference for the noise reproduction in simulated diffraction patterns.

1.2. Addition of Noise to the Simulated Diffraction Patterns

Synthetic noise was added to the calculated scattering intensities, at a level that closely matches the typical noise observed in routine experiments performed at the reference beamline. The addition was performed as follows:

$$I_{noisy,i} = I_{simu,i} + noise(I_i, c) = \frac{f(cI_{simu,i})}{c}$$

Where the $\boldsymbol{I_{simu}}$ are the simulated noise-less intensities. The function $f(x)$ returns a random number belonging to the Poisson distribution generated by its argument x. The noise level is tuned by the parameter c (higher c means less noise). To perform this calculation, the Random Poisson subroutine, available in the CrysFML repository (https://Code.Ill.Fr/Scientific-Software/Crysfml), was used and integrated into a locally developed Fortran code.

For simulated patterns, the S/N vector previously defined can be calculated directly as follows.

$$S/N_{simu,i} = \frac{signal_i}{\sigma_i} = \sqrt{c}\frac{max[\boldsymbol{I_{simu}}] - min[\boldsymbol{I_{simu}}]}{\sqrt{I_{simu,i}}}$$

The standard deviation σ_i of Poisson distribution is expressed by the square root of the ratio between the scattering intensity $I_{simu,i}$ and the scale c.

The optimal c value can be found by optimizing the difference between the ***S/N*** trace derived from the reference experimental data and the ***S/N*** trace derived from the simulation. This can be conveniently accomplished using only $\boldsymbol{S/N_{simu}}$ calculated at $c = 1$, by minimizing the value of φ by tuning the c parameter, without the need of multiple recalculations of $\boldsymbol{S/N_{simu}}$. The optimized c value can then be used for the final simulation, having a correct level of noise in respect to the experimental reference.

$$\varphi = \sum_i \left(S/N_{exp,i} - \sqrt{c} \cdot S/N_{simu,i}(c = 1)\right)^2$$

Noticeably, the minimum value of φ might not be optimal in case the estimation of the ***S/N*** values suffers from reliability issues. This was indeed the case for the RAPDF

setup, which typically provides patterns with very low noise levels. In this case, a visual adaptation of the c value was performed.

The c values determined for the RAPDF and MS-X04SA setups were respectively 45 e 0.85

The final SNR (signal/noise) were computed from the DSE simulations as $SNR = (\sum_{i=1}^{N} I_{simu}^2 / \sum_{i=1}^{N} \sigma_{simu}^2)^{1/2}$, and are reported in Table S1 for the two reference experimental setups.

2. CdSe Atomistic Models

In this work the reference CdSe model was adapted from a previously optimized one,[3] in order to resemble features common for CdSe Quantum Dots (QDs). Starting from this reference model, all the others were developed to selectively investigate the structural and microstructural features discussed in the main text.

Table S2. Mass-based average sizes and size distributions of the atomistic models described in sections 3.2-3.3 of the main text.

Description	D_{eq} (nm)	σ / D_{eq} (%)	L_{ab} (nm)	σ / L_{ab} (%)	L_c (nm)	σ / L_c (%)	AR (L_c / L_{ab})
Reference model	5.2	5.8	4.7	6.9	3.5	10.7	0.7
Small average size	2.9	7.6	2.6	10.5	1.9	10.4	0.7
Large average size	8.0	5.6	7.2	6.5	5.3	10.5	0.7
Large size dispersion	5.2	23.4	4.8	28.5	3.6	42.6	0.8
Spherical equivalent	5.2	7.0					
Plate-like morphology	5.2	6.5	7.7	7.1	1.3	14.7	0.2
Rod-like morphology with growth along the [001] direction	5.2	5.7	3.0	6.6	8.4	10.9	2.8
Rod-like morphology with growth along the [111] direction	5.2	5.8	3.0	6.6	8.4	11.0	2.8

All faulted models share the same size and shape of the reference model (Table S2).

Table S3. Stacking faults parameters used for the defective atomistic models described in sections 3.4 of the main text.

Description	α (cc→c)	β (hc→c)	γ (hh→c)	δ (ch→c)	SF (%)
Low SF %	0.95	0.95	1.00	0.96	6.2
Medium SF %	0.75	0.75	1.00	0.80	23.9
High SF %	0.54	0.54	1.00	0.55	39.8
Only ISF with SF %	0.85	0.85	1.00	0.00	24.7
Only GF with average SF %	0.57	1.00	1.00	1.00	22.8

3. Calculation of G(r)

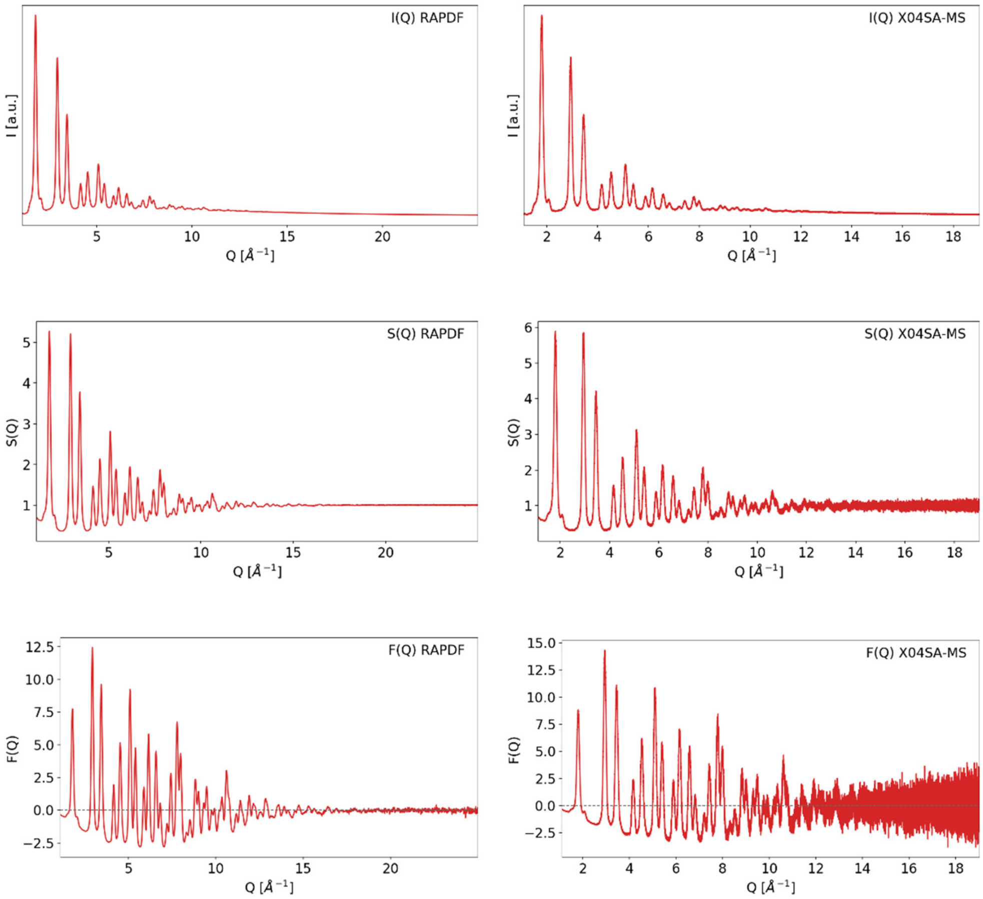

Figure S1. I(Q) (top), S(Q) (middle) and F(Q) (bottom) calculated for both the RAPDF (left) and the MS-X04SA (right) setups.

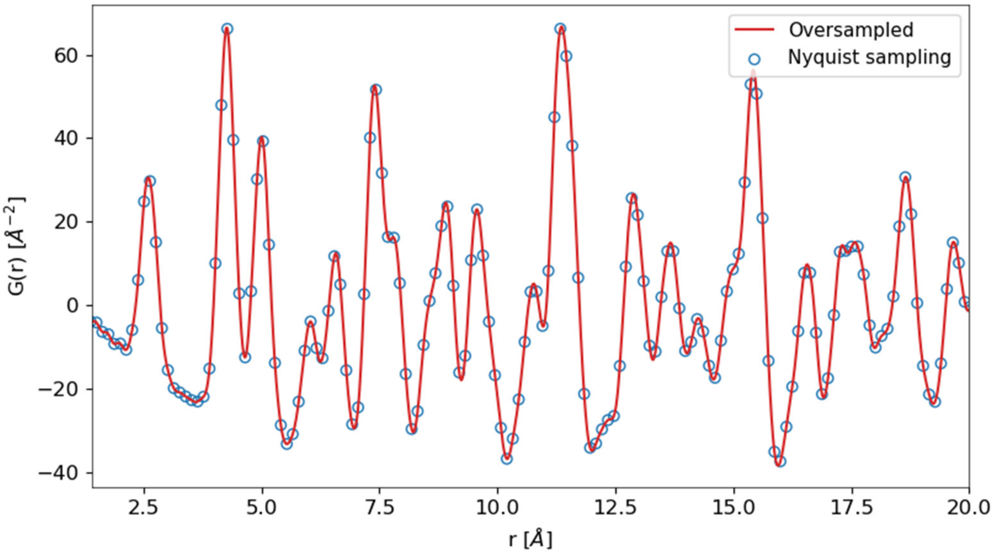

Figure S2. Comparison between the oversampled G(r) (red line) and the one obtained according to the Nyquist-Shannon theorem (blue markers).

4. Limitations on Average Size Determination Using the RAPDF Setup.

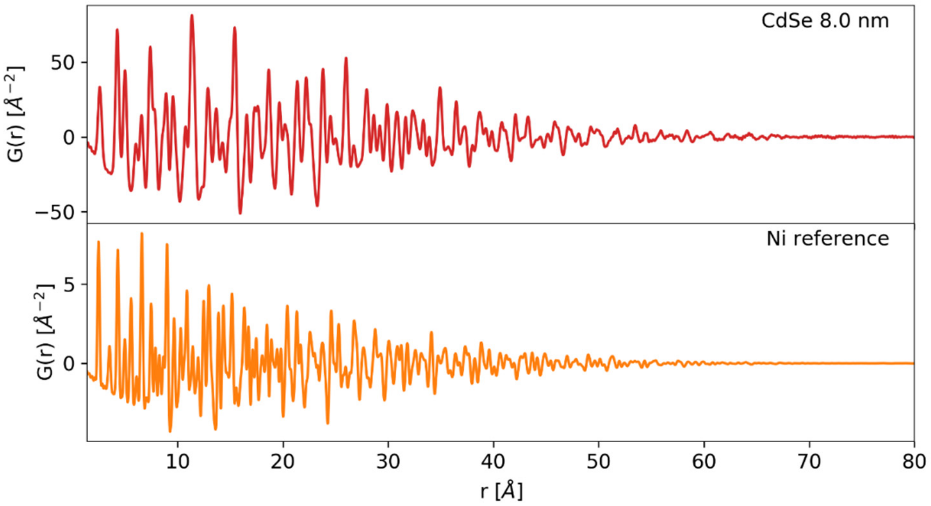

Figure S3. Comparison between the simulated CdSe G(r) trace, computed using an average spherical diameter of 8.0 nm (red line), and the experimental G(r) obtained from the reference polycrystalline Ni experimental data, collected using a RAPDF setup.

5. Effect of High SF% in the Case of Growth Stacking Faults.

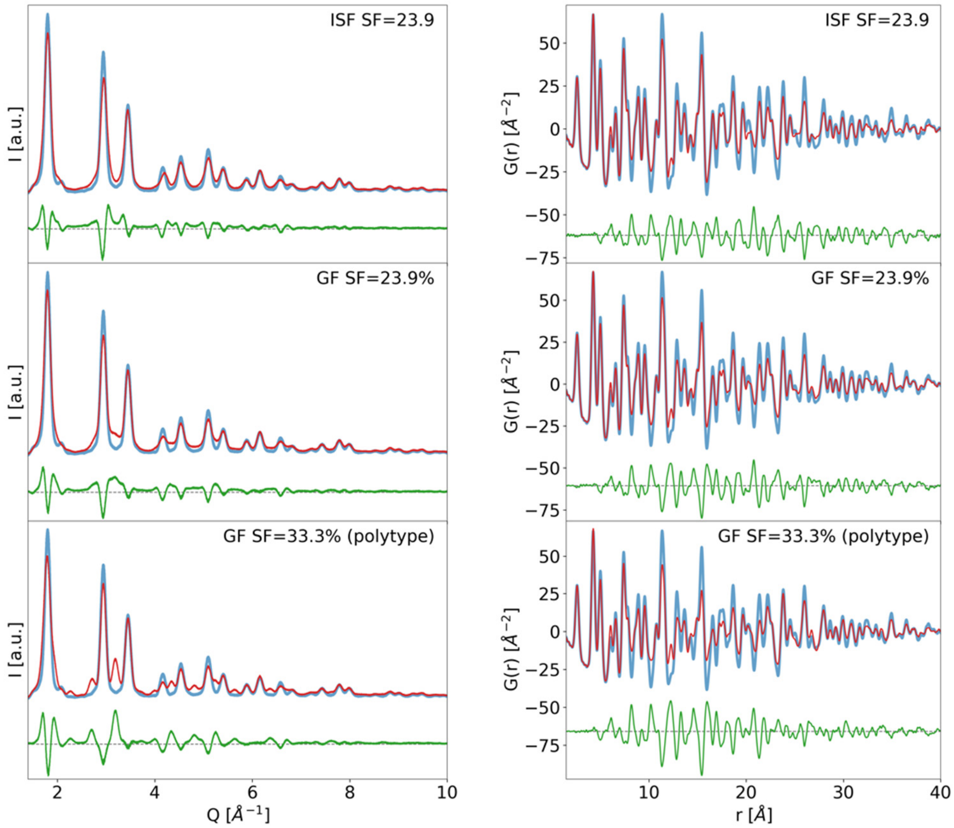

Figure S4. Comparison between simulated I(Q) (left, MS-X04SA setup) and G(r) (right, RAPDF setup) for the CdSe models with intrinsic stacking faults (ISF) only (SF% = 23.9%, top), growth faults (GF) only (SF% = 23.9%, middle), and GF only at SF% = 33.3% (bottom). All traces are plotted against the reference CdSe model, with ZB structure (SF% = 0%).

The case of the sample having only GF, at a SF% = 33.3%, corresponds to a different polytype (not wurtzite nor sphalerite). This polytype consists of the repeating stacking sequence …*cch*… and can be referred as the 6H$_1$ polytype using the Ramsdell notation. This explains why a system with only GF, with a SF% approaching 30%, shows additional peaks in reciprocal and real space, in comparison to the case with ISF only.

References

1. Chupas, P.J.; Qiu, X.; Hanson, J.C.; Lee, P.L.; Greya, C.P.; Billinge, S.J.L. Rapid-Acquisition Pair Distribution Function (RA-PDF) Analysis. *J. Appl. Cryst.* **2003**, *36*, 1342–1347.
2. Bergamaschi, A.; Cervellino, A.; Dinapoli, R.; Gozzo, F.; Henrich, B.; Johnson, I.; Kraft, P.; Mozzanica, A.; Schmitt, B.; Shi, X. The MYTHEN Detector for X-Ray Powder Diffraction Experiments at the Swiss Light Source. *J. Synchrotron Radiat.* **2010**, *17*, 653–668.
3. Moscheni, D.; Bertolotti, F.; Piveteau, L.; Protesescu, L.; Dirin, D.N.; Kovalenko, M.V.; Cervellino, A.; Pedersen, J.S.; Masciocchi, N.; Guagliardi, A. Size-Dependent Fault-Driven Relaxation and Faceting in Zincblende CdSe Colloidal Quantum Dots. *ACS Nano* **2018**, *12*, 12558–12570.
